# Subjective and Electroencephalographic Sleep Parameters in Children and Adolescents with Autism Spectrum Disorder: A Systematic Review

**DOI:** 10.3390/jcm10173893

**Published:** 2021-08-30

**Authors:** Maria Giuseppina Petruzzelli, Emilia Matera, Donatella Giambersio, Lucia Marzulli, Alessandra Gabellone, Anna Rosi Legrottaglie, Anna Margari, Lucia Margari

**Affiliations:** 1 Department of Basic Medical Sciences, Neuroscience and Sense Organs, University of Bari “Aldo Moro”, 70121 Bari, Italy; maria.petruzzelli@uniba.it (M.G.P.); lucia.marzulli@uniba.it (L.M.); margarianna2@gmail.com (A.M.); 2 Department of Biomedical Sciences and Human Oncology, University of Bari “Aldo Moro”, 70121 Bari, Italy; dona.215@hotmail.it (D.G.); alessandra.gabellone@uniba.it (A.G.); lucia.margari@uniba.it (L.M.); 3 University Hospital “Policlinico di Bari”, 70121 Bari, Italy; rosy.legrottaglie@gmail.com

**Keywords:** autism spectrum disorder, sleep, electroencephalography, polysomnography, neurodevelopmental disorders, CSHQ, sleep macrostructure, sleep microstructure

## Abstract

Background: Sleep problems have commonly manifested in children and adolescents with autism spectrum disorder (ASD) with a complex and multifactorial interaction between clinical and etiological components. These disorders are associated with functional impairment, and provoke significant physical and mental affliction. The purpose of this study is to update the existing literature about objective and subjective sleep parameters in children and adolescents with ASD, extrapolating information from polysomnography or sleep electroencephalography, and sleep related questionnaires. Methods: We have conducted a systematic review of case-control studies on this topic, performing a web-based search on PubMed, Scopus and the Web of Science databases according to the Preferred Reporting items for Systematic Review and Meta-analyses (PRISMA) guidelines. Results: Data collected from 20 survey result reports showed that children and adolescents with ASD experienced a higher rate of sleep abnormalities than in typically developing children. The macrostructural sleep parameters that were consistent with subjective parent reported measures unveil a greater percentage of nighttime signs of insomnia. Sleep microstructure patterns, in addition, pointed towards the bidirectional relationship between brain dysfunctions and sleep problems in children with ASD. Conclusions: Today’s literature acknowledges that objective and subjective sleep difficulties are more often recognized in individuals with ASD, so clinicians should assess sleep quality in the ASD clinical population, taking into consideration the potential implications on treatment strategies. It would be worthwhile in future studies to examine how factors, such as age, cognitive level or ASD severity could be related to ASD sleep abnormalities. Future research should directly assess whether sleep alterations could represent a specific marker for atypical brain development in ASD.

## 1. Introduction

Autism spectrum disorder (ASD) is a neurodevelopmental condition characterized by impairment in social interaction and communication, and by restricted, repetitive patterns of behaviors, interests, or activities [1]. ASD covers a group of complex and heterogeneous clinical situations with different levels of severity according to both the core symptoms and the frequent comorbidity with other neurodevelopmental disorders and medical conditions. Co-occurring clinical conditions generally endure from childhood to adolescence [2], worsening the health-related quality of life for both children and families.

Sleep disorders represent one of the most common co-occurring conditions in individuals with ASD and are a frequent occurrence among children and adolescents, with an estimated range from 11% to 37% of the population [3,4,5,6,7].

Previous research on parental reports and objective measurements taken from actigraphy and polysomnography have shown that ASD children are more prone to sleep problems than children with other neurodevelopmental disorders or children with typical development [8,9]. It is estimated that a range of 40% to 93% of the population of ASD children suffer from sleep disorders [4,7,8,10,11].

As far as our knowledge is concerned, the first meta-analysis examining objective and subjective measurements of sleep abnormalities in children with ASD was published in 2018 by A. Diaz Roman [12]. When compared with typically developing children, the author came upon patients with ASD presenting significant sleep impairments based on subjective and objective parameters. Further to this, the author noticed some congruities among similar parameters within objective studies, probably due to the different results obtained by polysomnography (PSG) or by actigraphy. In addition, he highlighted that the objective and subjective measurements were not related to each other, suggesting a complementary reading of information.

In recent years, an increasing number of studies have reported mixed results on objective and subjective measurements of sleep abnormalities in ASD patients [9,13,14,15,16,17,18]. The high level of heterogeneity across different studies may be related to biological, social, psychological and environmental factors influencing sleep problems in ASD [19].

The reciprocal relationship between ASD and sleep disorders is likely to affect a complex bidirectional association between neurobiological and environmental risk factors [20]. Therefore, it is better to raise awareness of the sleep architecture and its electrophysiological and microstructural features in ASD patients, to improve the knowledge on shared mechanisms in neural plasticity and brain functioning [11,21].

Research findings over the past two decades have supported a link between sleep states and synaptic plasticity. The term ‘sleep’ is defined as a periodic suspension of the state of consciousness, characterized by the slowing of the neurovegetative functions and by the partial interruption of the sensorimotor relationships of the subject with the environment, essential for brain restoration. The brain state is organized in cycles with two alternating phases: non-rapid eye movement (NREM) sleep, also termed slow-wave sleep (SWS), and rapid eye movement (REM) sleep. SWS is hallmarked by high-amplitude, slow oscillations (less than 1 Hz) and sleep spindles (0.5–2 s bursts of 10–16 Hz), while REM sleep is dominated by low-amplitude, wake-like fast oscillatory EEG activity (4–11 Hz) [22].

Recent progress showed that both synapse strengthening and weakening occurs across sleep, highlighting the function of sleep in regulating cortical synaptic plasticity. Different states of sleep affect different aspects of synaptic structural remodeling after the experience, so that memory consolidation and the retention of information can be disrupted by sleep deprivation or reduced sleep quality. This sleep-dependent synaptic plasticity plays an important role in neuronal circuit refinement during development and after learning [7,22,23].

It has been demonstrated that poor sleep hygiene could have negative impacts on children’s attention span, mood regulation and behavior. For this reason, sleep alteration during developmental ages may be considered as directly related to atypical brain development in many different neurodevelopmental disorders, as well as an additional risk factor for cognitive and behavioral impairment [21].

The aim of this study is to conduct a systematic review of case-control studies examining objective and subjective sleep measurement in ASD children and adolescents. Our quest is based on measuring sleep architectural parameters recorded through PSG or sleep electroencephalography (EEG) in order to reduce interpretative bias deriving from the comparison of electroencephalographic data and actigraphic measurements. Sleep architectural data were divided in macrostructural and microstructural parameters. Macrostructural parameters consist of: time in bed (TIB), sleep period time (SPT), total sleep time (TST), sleep latency (SL), sleep efficiency (SE), wakefulness after sleep onset (WASO%), percentage of rapid eye movement sleep (REM%), rapid eye movement sleep latency (RL), percentage of non-rapid eye movement stage 1 (N1%), stage 2 (N2%) and slow wave sleep (SWS%). Microstructural parameters instead were assessed with the scoring of cyclic alternating pattern (CAP), spectral analysis, and EEG band power and the detection of sleep electrophysiological features, such as sleep spindles, k-complex, and Mu rhythm.

In the hypothesis that an intrinsic cause of insomnia in ASD may be related to differences in brain wave organization and maturational development, the identification of microstructural EEG alterations may help to speculate on underlying pathophysiological mechanisms of the disease that alters sleep behaviors and sleep quality. Our role consists of collecting information on microstructural sleep abnormalities in children with ASD and at the same time identifying specific EEG biomarkers of atypical brain organization in ASD.

## 2. Methods

### 2.1. Study Selection Criteria

A web-based systematic review was performed on PubMed, Scopus and on the Web of Science databases, based on Preferred Reporting items for Systematic Review and Meta-analyses (PRISMA) guidelines [24]. The search terms were: “ASD” OR “Autism” OR “Autism spectrum disorder” OR “Asperger” OR “autistic disorder” OR “autis*” AND “sleep” OR “sleep disorder” OR “sleep disturbance” OR “sleep stages” AND “EEG” OR “electroencephalogram” OR “electroncephalogra*” OR “polysomnography” OR “polysomnogra*” OR “slow wave activity” OR “slow oscillations” OR “delta activity” OR “theta activity” OR “sleep spindles” OR “sigma activity” OR “cyclic alternating pattern” OR “sleep architecture” OR “sleep microstructure” OR “sleep oscillations”.

After the removal of duplicate records, papers were independently assessed based on the title and abstract by three authors (DG, LM and AG). Decisions regarding inclusion/exclusion by each reviewer were recorded and any discrepancies or conflicts between reviewers were resolved through discussion and were on the side of over-inclusion at this stage.

Articles had to meet the following inclusion criteria:

(a) original observational studies published between January 2000 and December 2020;
(b) studies conducted on children and/or adolescents aged between 0 and 18 years old;
(c) clear indication of the procedure followed to arrive at the formal diagnosis of ASD, conducted through clinical judgment alone or with the support of standardized diagnostic tools in accordance with the criteria of the DSM-IV or DSM-5;
(d) studies reporting objective sleep parameters measured using sleep EEG or polysomnography and/or subjective sleep parameters from any sleep questionnaire;
(e) English language was mandatory.

Furthermore, papers were excluded if they were:

(a) reviews, meta-analysis articles, book chapters, meeting abstracts or case report/case series articles;
(b) studies on subjects with autistic traits or in which the diagnostic process and/or tools were not clearly specified;
(c) clinical or human research studies (i.e., not animal models);
(d) papers referring to ASD related to specific genetic mutations or syndromes.

The full-text screening followed the same criteria. A senior author (MGP) intervened in any case of disagreement. Three reviewers (DG, LM and AG) independently extracted and cross-checked data for the included studies: first author, publication year, ASD assessment and diagnostic criteria, type of ASD diagnosis, study sample features (number of participants, male: female ratio, mean age (±sd) and/or range of age), inclusion/exclusion criteria, sleep EEG or PSG recording characteristics, and type of sleep questionnaire, main results with the associated statistical significance.

### 2.2. Results Analysis

This review includes a descriptive analysis of the results achieved by the selected studies. Data were narratively presented in the main text in an aggregated and summarized form and for a clearer understanding, *p*-values, effect size, and correlation coefficients where applicable, were reported for statistically significant findings only. Due to the large amount of data collected on sleep macrostructural measures and on subjective findings, we reported their means and standard deviations (or median with minimum and maximum when appropriate) in supplementary materials as follows: for macrostructural measures see Table S1, for microstructural measures see Table S2, and for subjective findings see Table S3.

## 3. Results

**Figure 1:**
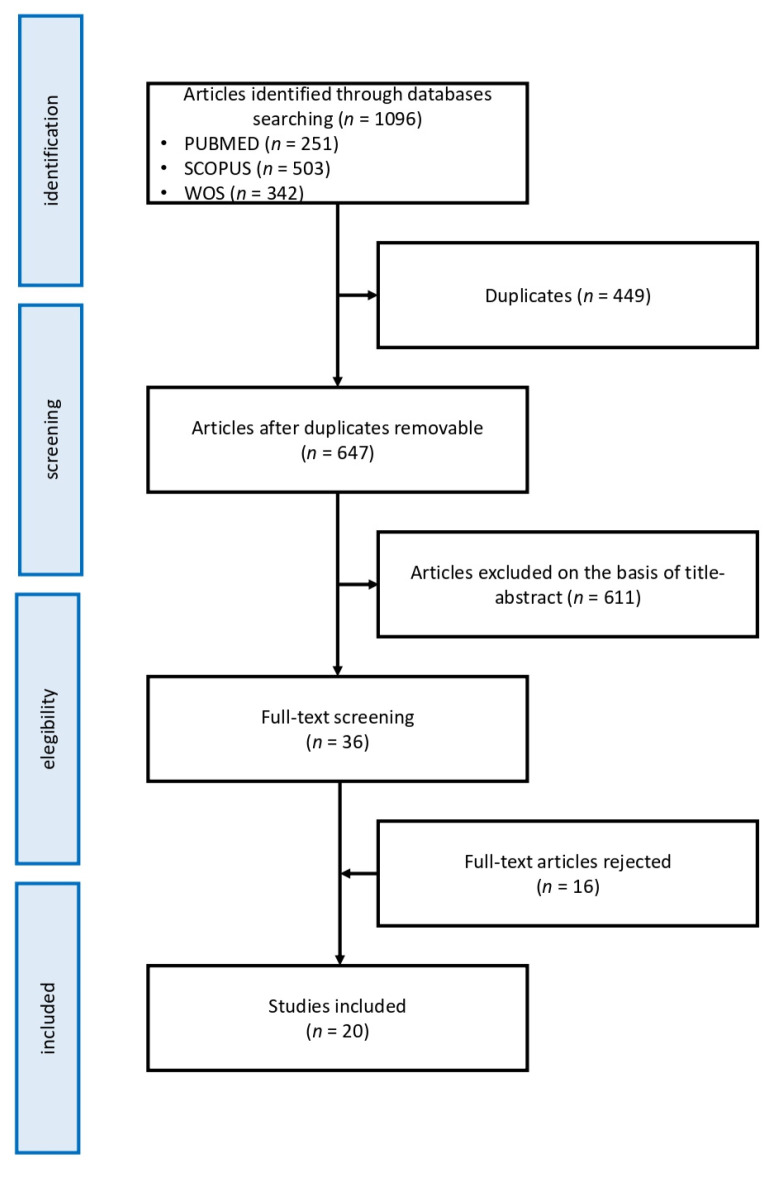
Study selection flow chart.

In conformity with our inclusion and exclusion criteria research, on a total number of 1096 surveys, 647 were retained as valid in accordance with the title and abstracts. Consequently, 36 potential pertinent full-texts were examined by two authors. The overall value was calculated using Cohen’s kappa coefficient, with a k value of 0.77 indicative of substantial agreement. After two studies were excluded based on a third author’s opinion, 20 of them were retained as valid. All the authors ascertained the quality of the included papers, and any disagreement was solved through consensus. Figure 1 shows the flow diagram of the study selection. Table 1 summarizes the studies’ features, Table 2 summarizes the main results of the identified studies.

### 3.1. Macrostructural EEG Parameters

Fourteen case-controlled research studies have analyzed sleep macrostructure parameters through overnight sleep evaluations [25,26,27,28,29,30,31,32,34,35,37,38,43,44]. They were performed using polysomnography and the test was conducted on two nights for a more precise value. Recordings were usually conducted in a sleep laboratory, with the exception of three studies [34,37,43] in which PSG was conducted in patients’ bedrooms. All recordings started at the patients’ usual bedtime and continued until spontaneous awakening. The following objective sleep parameters, generally scored according to the standard criteria by Rechtschaffen and Kales [45], were evaluated: time in bed (TIB: time spent in bed), sleep period time (SPT: time passed between the sleep onset and sleep end), total sleep time (TST: time spent sleeping, that is SPT minus time awake), sleep latency (SL: time from lights out to sleep onset), sleep efficiency (SE: the ratio of TST to TIB), wakefulness after sleep onset (WASO%: percentage of SPT spent in wakefulness after sleep onset), percentage of REM sleep (REM%), REM latency (RL: time occurred from sleep onset to the first REM sleep epoch), percentage of NREM stage 1 (N1%), stage 2 (N2%) and slow wave sleep (SWS%).

#### 3.1.1. ASD Children vs. TD Children

Available literature demonstrated a higher rate of alterations of the sleep macrostructure in ASD children compared with typically developed (TD) children (Table S1). However, results of the studies differed with respect to each analyzed parameter as shown in Tables S1 and S4.

Six studies analyzed TIB, with a significantly shorter value detected in ASD patients in three of them (*p* < 0.01 [25], *p* < 0.044 [27], *p* = 0.02 [44]).

TST is one of the most examined parameters. Six studies reported a statistically significant reduction of TST in ASD patients (*p* < 0.02 [25], *p* < 0.007 [27], *p* < 0.001 [31], *p* = 0.004 [32], *p* < 0.05 [43], *p* = 0.03 [44]), and, accordingly, SPT was over fifty minutes shorter in ASD patients than in TD (*p* < 0.01 [25], *p* < 0.014 [27].

A statistical difference was found by Giannotti et al., Lambert et al., and Maski et al. with respect to SL (*p* < 0.001, *p* = 0.02, *p* = 0.01, respectively), with a higher value in ASD patients [31,35,37].

Many studies have shown a lower SE value in ASD patients [25,27,30,35,38,44], although only Maski et al. found a statistically significant difference (*p* < 0.001) [37].

Contrasting results were obtained regarding WASO [25,27,29,31,32,35,37,43,44], but the only two significant findings showed a greater value of WASO in ASD patients (*p* < 0.001 and *p* = 0.02, respectively) [31,37].

If ASD children and adolescents took a longer time to reach REM sleep is unclear, since studies showed contrasting results.

A lower REM% has been found by most of the studies, with significant results in some of them (*p* = 0.002 [30], *p* < 0.001 [32], *p* = 0.049 [37], *p* = 0.007 [44]).

One study demonstrated an overall reduction of NREM% in ASD children compared to TD (*p* < 0.05) [43], while contrasting results were obtained for “slow wave sleep” (a lower SWS percentage in *p* = 0.026 [35], *p* = 0.007 [38], *p* = 0.001 [44], and a higher SWS percentage in Buckley, *p* = 0.001 [32]).

#### 3.1.2. ASD Children vs. Developmentally Delayed Children

A comparison was conducted between ASD children and non-ASD children affected by other developmental delays (DD) in two papers [25,32]. When compared with children with X-fragile syndrome, ASD samples showed a significantly reduced REM latency and N1 percentage (*p* < 0.01 and *p* < 0.05 respectively) [25]. In addition, ASD samples showed a lower TIB, SPT, TST, SE, REM% and N2% value, although no substantial differences were found [25].

Buckley et al. compared ASD children with DD children, finding a significantly reduced TST and REM percentage (*p* = 0.001, *p* < 0.001) and a prolonged RL (*p* = 0.012). Furthermore, the SWS percentage was significantly higher in ASD children than in DD (*p* < 0.001). ASD differed from the DD sample in a non-significant way for the other evaluated parameters (% N1 and N2, SE, WASO, SL) [32].

#### 3.1.3. Differences in ASD Subgroups

In sleep architecture, some dissimilarities were found within the ASD sample. Giannotti compared two ASD subgroups, depending on the history of autistic regression. He discovered that ASD children with regression had a significantly reduced TST value compared to ASD children without regression (*p* < 0.001) [31]. ASD children without any history of regression showed higher SE, lower WASO, and lower SL (*p* < 0.001) than ASD children with regression.

When ASD children were compared with children with Asperger syndrome, none of the results were significantly different. In particular, ASD children showed no shorter TIB, TST, SPT, WASO, SL and RL than Asperger ones [28]. They also noticed a higher SE and a higher percentage of REM and N1 sleep, with a shorter percentage of N2 and SWS, but none of these results reached statistical significance.

### 3.2. Microstructural EEG Parameters

Ten studies focused on different electrophysiological features of the microstructure of sleep [27,28,31,33,35,42,43].

Sleep spindle parameters (number, duration, density) were examined by two different research groups [33,41]. Tessier et al. found that, in comparison with the TD group, the ASD group displayed lower stage two sleep spindle density at the Fp2 electrode for the total night (*p* < 0.05) and the second quarter of the night (*p* < 0.05) and shorter sleep spindle duration at the Fp1 electrode for the second quarter of the night and the third quarter of the night (*p* < 0.05) [33]. Farmer et al. found a significantly lower spindle density in children with ASD when compared with non-ASD children with other developmental delays (*p* = 0.017) and with typical development (TD) (*p* < 0.0001) [41]. The spindle duration was significantly shorter in the ASD group only when it was compared with the TD group (*p* = 0.006). Spindle frequency was also lower in ASD children in comparison with the other two groups, but not in a significant way.

Three papers examined cyclic alternating pattern (CAP) parameters (total CAP rate, average CAP cycle duration, number of CAP cycles, percentage and duration of each phase A subtype (A1%, A2%, A3%, and A2e3%) [27,28,31]. Miano and Giannotti showed a significantly decreased percentage of A1 (*p* < 0.0004, *p* < 0.001 respectively), and an increased percentage of A2 (*p* < 0.006, *p* < 0.01, respectively) and A3 (*p* < 0.02, *p* < 0.001, respectively) in patients with autism compared with TD controls [27,31]. Furthermore, Miano et al. found a lower CAP rate during SWS in ASD children than in TD (*p* < 0.02) [27]. Among ASD patients, children with regressive autism had a reduced CAP rate in N1 and N2 sleep compared with children without regression (*p* < 0.01) [31]. Conversely, an increased CAP rate during SWS in subjects with Asperger syndrome vs. children with autism was found (*p* < 0.02) [28].

“Band power” is the power of the signal measured in µV2/Hz, of the main five EEG frequency bands (delta, theta, alpha, beta and gamma); absolute band power reflects EEG activity in one frequency band independently of the other bands, while relative band power reflects the EEG activity and the relationship with other bands [33,36,42]. Sahroni et al. performed a medication-induced short sleep EEG test and found that in the ASD group there was a greater absolute band power of alpha (*p* < 0.03) and theta (*p* = 0.0379) and a greater relative band power of delta (*p* = 0.0379); alpha bands have the major power, especially in the frontal region. Instead, in the TD group there was a greater absolute band power of gamma (*p* < 0.04) and a greater relative band power of gamma (*p* < 0.04) and beta (*p* < 0.04) [36].

Similarly, Tessier et al. found a higher theta activity in their high functioning ASD patients aged 6–13 years than in TD children in a parietal derivation (*p* < 0.05) [33]. Page et al. used a 124- or 128-channel high density EEG net to examine EEG band power during a nap with an average duration of 78 min in children 13–30 months old with ASD. They detected differences in the NREM spectral features between TD and ASD, with a significant decrease of fast theta power in the temporo-central cluster and a significant increase in beta power in the right temporo-occipital cluster (*p* < 0.05) [42].

Finally, Vite et al. analyzed the Mu rhythm, defined as an arc-shaped rhythm, with an acute negative and rounded positive component, within the frequency of 8–13 Hz, expressed most intensely in somatosensory regions, specifically in C3 and C4 [40]. They observed that in comparison with the TD group, children with a diagnosis of “ASD-level 1” showed during sleep fewer Mu segments in each of the phases of sleep. In addition, the ASD group showed a significantly higher peak in the power spectrum of the Mu rhythm in C3 derivation (left hemisphere) than in the TD group and significantly lower in C4 (right hemisphere) (*p* = 0.003, *p* = 0.023, respectively).

### 3.3. Subjective Parameter

A qualitative characterization of sleep was performed in sixteen papers [26,27,28,29,30,31,32,33,34,35,37,38,39,42,43,44], most of them using the Children’s Sleep Habit Questionnaire (CSHQ) [26,29,31,32,35,37,39,43,44], a parent-report standardized screening instrument designed primarily for surveying sleep habits and sleep disturbances [46]. It includes 45 items exploring seven sleep domains that encompass the major clinical sleep complaints based on common clinical symptom presentations of the most prevalent pediatric International Classification of Sleep Disorders (Bedtime resistance, Sleep onset delay, Sleep duration, Sleep anxiety, Night wakings, Sleep disorder Breathing, Daytime sleepiness, Parasomnias). Comparing ASD and TD, an overall higher total CSHQ score in ASD was obtained (Table S3), with significant results found by Goldman et al. (*p* < 0.05) [29], Maski et al. (*p* < 0.001) [37] and Fletcher et al. (*p* < 0.001) [43].

ASD patients showed significantly higher bedtime resistance scores according to Giannotti, Maski and Aathira and their colleagues (*p* < 0.001, *p* = 0.03, *p* < 0.001, respectively) [31,37,39].

The CSHQ sleep onset delay subscale was significantly higher in ASD than in TD (*p* < 0.01 [29], *p* < 0.001 [31], 0.02 [37], *p* = 0.01 [39]). Only one study identified a lower sleep duration report in ASD, while the others pointed out an equal or higher sleep duration, with significant findings for Goldman’s, Giannotti’s and Maski’s samples (*p* < 0.05, *p* < 0.001, *p* = 0.04, respectively) [29,31,37].

All studies identified higher sleep anxiety levels in ASD children and adolescents than in TD controls, but significance was reached in two of them (*p* < 0.001 [37], *p* = 0.002 [39]). Studies by Lambert and Aathira revealed a lower number of night wakings in ASD individuals, but without significance [35,37]. Conversely, a higher night walking score was obtained in other studies (*p* < 0.01 [29], *p* < 0.001 [31]). With the exception of Lambert et al. [35], all studies found higher scores for disordered breathing in ASD, but significance was reached in only one study (*p* = 0.034) [39]. As a consequence of disrupted sleep, all studies described higher scores on the daytime sleepiness subscale, significant for three analyses (*p* < 0.05 [29], *p* < 0.02 [37], *p* < 0.001 [39]). The parasomnia subscale was found to be associated with higher scores for all studies investigating it, reaching statistical significance in three cases (*p* < 0.05 [29], *p* = 0.02 [37], *p* < 0.001 [39]). Parental concern about sleep, investigated only by Aathira et al., was found to be higher in ASD than in TD (*p* < 0.001) [39].

Buckley et al. simply reported the median wake time for ASD (6:17 a.m.), DD (6:45 a.m.), and TD (6:46 a.m.) [32], which differed from those given by Aathira et al. (7:28 a.m. for ASD, 7:05 a.m. for TD) [39]. However, in Aathira et al. the time of sleep onset was indicated (10:55 p.m. for ASD, 10:13 p.m. for TD) [39]. Three studies used a regular sleep–wake schedule for 14 days and a self- or parent-reported sleep diary [33,34,38] and one study [42] analyzed only the naptime and the duration of wakefulness before the nap, finding no significant differences between ASD and TD children.

Only three studies used a sleep questionnaire originally created for the purposes of their investigation [27,28,30]. Bruni et al. added the Pediatric Daytime Sleepiness Scale (PDSS) to evaluate the relationship between daytime sleepiness and school-related outcomes [47]. The results are mostly overlapping with those of the CSHQ.

Miano et al. and Bruni et al. identified a shorter sleep duration in the ASD sample and, in addition, a high prevalence of several other sleep problems in ASD children (i.e., bedwetting, rhythmic movements while falling asleep, sleep restlessness, daytime somnolence, falling asleep at school) [27,28].

Ming et al’s sleep questionnaires included questions on the current and past history of different sleep problems, but they mainly focused their attention on parasomnias [30]. The most common parasomnia found in the ASD population was the Disorder of Partial Arousal, with an estimated prevalence of 28.9% when evaluated by questionnaires vs. 29.4% when estimated by PSG [30].

## 4. Discussion

This systematic review provides the status of knowledge about the occurrence of sleep problems in children and adolescents with ASD, considering objective parameters from sleep EEG or polysomnography and subjective parameters from sleep questionnaires. Collected data validate literature evidence that, patients with ASD experience a greater rate of qualitative and quantitative sleep disturbances than children and adolescents with typical development.

Considering that sleep problems in ASD are likely to recognize a multifactorial etiology, many possible neurobiological, medical, behavioral, and cultural mechanisms have to be considered in order to correctly understand the meaning of objective and subjective sleep measurement findings.

The most significant findings from macrostructural sleep parameters were shorter TST, higher SL and a higher number of WASO. These data, consistent with previous literature [12,48], pointed out that ASD patients, compared with TD children, take a longer time to fall asleep, sleep less, and they have a greater number of awakenings after sleep onset than TD children, each one corresponding to a higher rate of nighttime symptoms of insomnia.

These objective findings were reasonably consistent with subjective parent reported measures. Total scores obtained by CSHQ and other questionnaires were higher in ASD when compared with TD children and adolescents, suggesting a higher rate of sleep problems. The parents’ perception of higher bedtime resistance, higher sleep onset delay and higher night waking activities supports the clinical hypothesis that ASD patients may have greater difficulties with initiating and maintaining sleep than TD children.

Sensory integration deficits, ritualistic or self-injurious behaviors, poor communication skills, and limited responsiveness to social cues can exacerbate bedtime resistance, prolonging the amount of time from lights turned off until the onset of any sleep stage, and interfere with sleep continuity. For example, repetitive patterns of behavior, such as going to bed and delayed sleep onset, particularly when ASD children are selectively absorbed by unusual activities during or around sleep (wearing particular pajamas, having a particular toy to play with, performing specific rituals etc.). ASD children with over-sensitivity may have a greater vulnerability to noise, light, and temperature, resulting in environmental discomfort and impairment of sleep quality. Moreover, the ASD phenotype and severity of core symptoms may negatively interfere with symptoms of insomnia, it also happens that a worse quality of sleep impacts daytime behavior problems and the adaptive skill development of ASD children worsens the quality of life of both ASD individuals and their caregivers [49,50].

Other qualitative features of sleep, detectable only through subjective measures, showed that ASD patients have more frequent parasomnias and higher sleep anxiety levels, such as being afraid to sleep in the dark or alone. These symptoms can be further related to having difficulty in initiating and maintaining sleep. The percentage of time spent in non-rapid eye movement (NREM) and REM sleep and the weaker slow wave activity (SWA) identified in some research [43,44] may be considered as additional macrostructural data making children with ASD uniquely vulnerable to sleep problems. SWA in fact, is an index of sleep pressure, defined as the “homeostatic response to sleep deprivation”, and its reduction could lead to several sleep disturbances, particularly sleep onset and sleep maintenance difficulties [44]. Underlying biological and behavioral rhythms may predispose to both extrinsic and intrinsic stressors that threaten sleep, making children with ASD uniquely vulnerable to sleep problems. We know that sleep is under both circadian and homeostatic control and that sleep time (percent time spent in wakefulness, REM and NREM sleep) across the lifespan is a function of age. Sleep regulatory mechanisms are not present at the time of birth, but they mature during development at different rates [51].

A biological connection between insomnia and ASD has been reported, considering that biological mechanisms and specific genes implicated in GABAergic inhibition, serotonergic transmission and dysregulation of the melatonin pathway are thought to be involved in both disorders [8,11]. Genetic and neurobiological findings highlighted the major role of synaptic and clock genes in the susceptibility to ASD [52]. Several lines of evidence suggest that melatonin could modulate neuronal networks by influencing both the strength and the circadian oscillation of neuronal transmission, while the NLGN/NRXN/ SHANK3 synaptic pathway could alter the clock and the circadian rhythms in individuals with ASD [51]. For example SHANK3, a high confidence ASD gene candidate, is an important modulator of sleep that may exert its effect through the regulation of circadian transcription factors [53]. Also mutations in the FMR1 and MECP2 genes were shown to alter the circadian rhythms and sleep/wake cycles of patients with Fragile-X or Rett syndrome, respectively [52,54].

One of the most relevant hypotheses regarding an intrinsic cause of insomnia in ASD involves possible differences in brain wave organization and maturation that can be identified by polysomnography [7].

Studies on sleep microstructure patterns, although fewer in number, may give more information about the bidirectional relationship between brain dysfunctions and sleep problems in ASD children. Some authors hypothesized a critical role for sleep EEG oscillations on neural plasticity in different neurodevelopmental disorders, considering the parallel maturational changes of brain and sleep features across development. A growing interest actually exists in studying the association between alterations in several sleep oscillations and NDDs, with the purpose of clarifying the specificity and the functional meaning of this association [21]. The papers we reviewed showed alterations of sleep spindles, CAP, band powers, and the Mu rhythm in ASD patients, but the meaning of these data should be interpreted with care, considering the paucity of numbers, and the clinical heterogeneity of studies’ samples.

Tessier and Farmer found a decrease of spindle density and duration in ASD children compared to controls, suggesting that this finding may be considered a biomarker of functional anomalies in brain maturation. Spindle activity, originated in the thalamic reticular nucleus, is considered as an electrographic landmark from waking to sleep transition, protecting sleep from being interrupted by external stimuli [33,41]. Sleep spindles exhibit an age-dependent pattern, and their developmental modifications are presumably related to the maturation of thalamic-cortical structures, so that spindles are believed to play an important functional role in sleep-dependent synaptic plasticity and memory consolidation [21,41,55].

Miano and Giannotti found a low CAP rate in ASD patients associated with a decreased A1 index during SWS and increased A2 and A3 indexes during light sleep, corresponding to markers of NREM sleep instability. In addition, the decrease in A1 CAP subtypes might play a role in the impairment of cognitive functioning in ASD patients [27,31]. According to this theory, a higher CAP rate was found in Asperger patients with a higher Intelligence Quotient (IQ). Several studies have focused on the importance of the relationship between CAP and cognitive performance in children with neurodevelopmental disabilities, showing peculiar CAP modifications in accordance with the degree of mental ability/disability.

The results about band powers may be indicative of brain dysfunction in ASD children, and the increase of alpha power in the frontal region, found by Sharoni, could be associated with the altered emotional response and emotional disorders in children with ASD [36].

On the other side, studies conducted on the theta band achieved opposite results, probably because of the different age groups in which the research was conducted. It is indeed known that EEG NREM spectral features vary across the first years of life [42,56]. However, it is interesting to note the finding of a less pronounced theta power in the centro-temporal region in ASD infants that may be linked to a reduced centro-temporal dopaminergic activity, suggested to be associated with socio-communicative impairment in autism [57].

Finally, the only study about the sleep ‘Mu rhythm’ demonstrated a lower activity of the Mu rhythm in ASD children, mainly in the right hemisphere. The abnormalities in Mu rhythm, which originates from pyramidal neurons in somato-sensorial regions, may reflect an abnormal activity of the mirror neuron system [58,59]. Studies analyzing the Mu rhythm during wakefulness in ASD children demonstrated a lack of suppression of Mu waves during the observation of a movement performed by other people, which indicated a reduction of the capacity of imitation, learning and the ability to understand others’ actions [60,61]. The presence of a Mu rhythm during the night showed that this rhythm is produced intrinsically, not only in response to external sensory stimulation, while the lower representation in the right hemisphere of ASD patients may demonstrate its higher impairment.

Due to disorder heterogeneity and methodological differences among studies, a unique pattern regarding microstructural sleep alterations in ASD has not been identified so far. These findings suggest that sleep EEG alterations may be a biomarker of intrinsic vulnerability to insomnia in ASD patients as well as a general hallmark of abnormal structural and functional brain maturation associated with the impairment of intelligence, memory and emotional processes [21,51,62].

### Limitations and Future Directions

The considerable limitations of the literature about sleep disorders in ASD stems from the scarcity of studies and, mainly, their heterogeneity. Methodologies for assessing sleep patterns and participants’ features differ widely among studies, contributing to achieving different conclusions that are only partially comparable. Moreover, studies often fail in providing information about the studied sample, such as concomitant medications, co-occurring disorders, circadian and environmental factors, or the cognitive level and behavioral profile that may potentially affect the quality of sleep. Some studies have excluded children with sleep disorders from the TD group, although, in most cases, only patients with breathing disorders and sleep apnea were excluded. This could be a bias that led the findings towards a worse sleep schedule in ASD. Other factors should be taken into account, for example: not all ASD children are able to tolerate EEG or PSG due to their sensory sensitivity, and sleeping in an unusual place, such as a laboratory setting, could worsen the cycle and quality of sleep in ASD patients [49,50]. Furthermore, considering the physiological maturational changes of sleep EEG patterns and sleep clinical features with growth, age ranges of enrolled patients should be restricted, and longitudinal studies should be conducted in order to better explore the relationship between sleep and neurodevelopment. Therefore, it would be worthwhile in future studies to fully characterize study samples, trying to correlate specific sleep abnormalities to specific ASD clinical phenotypes. Further research should opt for homogeneous sleep characteristics in the ASD and TD groups to better scrutinise them in terms of sleep disorders. Furthermore, current literature did not clarify whether sleep alterations could represent a specific marker for atypical brain development in ASD, so future studies with rigorous longitudinal design will be needed.

## 5. Conclusions

As awareness of the critical role of sleep for healthy physical and mental development has grown up, and the study of sleep in neurodevelopmental disorders and particularly in ASD has received more attention. Despite some limitations, the reviewed studies clearly show how objective and subjective sleep abnormalities are recognized in children and adolescents with ASD. These abnormalities can be identified by the polysomnographic analysis of macro- and/or microstructural features of sleep and by the collection of clinical information by parents. Therefore, clinicians should always assess sleep features in the ASD clinical population, given that the identification of sleep difficulties could have implications in the choice of treatment strategies.

Further studies are needed to understand the integrating mechanistic theory of the overlap between sleep disorders and ASD, although there has been progress in understanding the biology underlying disorders, with the involvement of genetic, medical, and behavioral factors. Lastly, whether specific sleep EEG patterns could represent a marker for atypical brain development in ASD is unclear and it needs an ad hoc design for future research.

## Figures and Tables

**Table 1:** Studies reporting findings on sleep EEG features and sleep subjective features in ASD patients.

Author, Year, Country	ASD Group					Control Group			PSG/EEG Features	Subjective Measures
	*n* (% Male)	Mean Age (Age Range)	Diagnosis	Diagnostic Tools	Inclusion/Exclusion Criteria	Type	*n* (% Male)	Mean Age (Age Range)		
Elia, 2000, Italy [25]	17 (100%)	10.36 years (5.7–16.8 years)	Autistic disorder (DSM-IV)	CARS	Inclusion criteria in AD patients: Diagnosis of AD according to the DSM-IV guidelines Exclusion criteria in AD patients: Interictal paroxysmal activity in the EEG Other pathological conditions (fragile X syndrome, epilepsy, cerebellar abnormalities at brain MRI) Drug period of 6 months preceding and during the study	fragile XTD	7 (100%) 5 (100%)	9.92 years (8.25–12 years) 9.22 years (7.17–11.58 years)	2 PSG with one adaptation night	-
Malow, 2006, USA [26]	21(85.7%)	- (4–10 years)	Autism, pervasive developmental disorder, Asperger’s disorder (DSM-IV)	ADOS	Inclusion criteria in ASD patients: Clinical diagnosis of ASD from psychologist or clinicians verified by the ADOS Exclusion criteria in ASD patients: Psychotropic medications History of epileptic seizures or mental retardation	TD	10 (80%)	(4–10 years)	2 consecutive nights of video monitoring combined with EEG and PSG with 21 EEG channels	Sleep diaries CSHQ
Miano, 2007, Italy [27]	31 (90.3%)	9.53 years (3.7–19 years)	Autistic disorder (DSM-IV)	CARS	Inclusion criteria in ASD patients: Diagnosis of autism according to DSM-IV and a score on the CARS > 30 Mental retardation Drug-free period for at least 2 weeks before the study began Exclusion criteria in ASD patients: Presence of neurological focal signs, seizures or paroxysmal EEG abnormalities Presence of craniofacial abnormalities, fragile X syndrome or other chromosome abnormalities Presence of obesity and no history of snoring	TD	18 (50%)	age-matched	Overnight PSG recording after one adaptation night. The PSG montage includes at least 3 EEG channels (including C3 and C4 in particular)	Sleep Questionnaire
Bruni, 2007, Italy [28]	10 autism (90%) 8 Asperger syndrome (87.5%)	autism: 11.9 ± 2.5 years (7–15 years) Asperger syndrome: 12.7 ± 2.6 years (7–15 years)	Autism (DSM-IV) Asperger syndrome (DSM-IV)	CARS	Inclusion criteria in patients with AS: Clinical diagnosis of AS verified by the ADOS Inclusion criteria in patients with autism: Diagnosis of autism according to DSM-IV and a score on the CARS > 30 Presence of mental retardation Exclusion criteria in patients with AS: History of serious physical health problems, epileptic seizures or mental retardation Presence of obesity or snoring Taking medications Exclusion criteria in patients with autism: Drug-free period from at least 2 weeks before the study began Presence of neurological focal signs, seizures or paroxysmal EEG abnormalities Other medical conditions associated with autism Obesity and history of snoring Exclusion criteria in control patients: Anamnesis of neuropsychiatric disorders Current axis I or axis II psychiatric disorders Complaints of sleep problems.	TD	12 (41.6%)	12.6 ± 3.7 years (7–15 years)	PSG overnight The PSG montage included at least 8 EEG channels (Fp1, Fp2, C3, C4, T3, T4, O1, O2) All recordings started at the patients’ usual bedtime and continued until spontaneous awakening.	Sleep Questionnaire PDSS
Goldman, 2009, USA [29]	27 psASD (88.9%) 15 gsASD: (93.3%)	PS: 5.8 years GS: 5.9 years	ASD (DSM-IV)	ADOS	Inclusion criteria in ASD children: Clinical diagnosis of ASD confirmed by the ADOS Exclusion criteria in ASD children: History of epilepsy Intellectual disability Psychotropic medications	TD	16 (75%)	6.9 years	Two consecutive nights of PSG with 21-channel EEG and actigraphy monitoring	PCQ CSHQ
Ming, 2009, USA [30]	23 (82.6%)	6 years (3–15 years)	autistic disorder, PDD-NOS, Asperger’s disorder (DSM-IV)	ASD ADI-R, ADOS-G	Inclusion criteria in ASD patients: Clinical diagnosis of ASD according to DSM-IV and confirmed by ADI-R and ADOS-G Complaints of sleep related problems Exclusion criteria in ASD patients: Presence of focal neurological signs Exclusion criteria in control patients: No known developmental or chronic illness with the exception of acute reactive airway disease and/or allergy in 7 children No history of neurological disorders and no acute illness including reactive airway disease or allergy at time of PSG study	TD	23 (65.2%)	5 years (3–12 years)	PSG for 2 consecutive nights with standard four channel EEG for PSG	Sleep Questionnaires
Giannotti, 2010, Italy [31]	22 NRegASD (75%) 18 RegASD (80%)	5.5 years 5.10 years	autistic disorder (DSM-IV)	ADI-R, ADOS-G	Inclusion criteria in autistic groups: Clinical diagnosis of autism according to the DSM-IV and confirmed by the ADOS-G and ADI-R NVIQ > 50 Exclusion criteria in autistic groups: Presence of other serious neurological, psychiatric, bipolar disorder or medical conditions Presence of obesity and history of snoring Altered hematological screening Presence of infections, inflammatory or allergic reactions for at least 6 months prior to the beginning of the study	TD	12 (75%)	5.8 years	Overnight PSG including at least 11 EEG channels (Fp1, Fp2, C3, C4, Cz, P3, P4, T3, T4, O1, O2) for 2 consecutive nights	CSHQ
Buckley, 2010, USA [32]	60 (82%)	4.81 years (2.24–13.11 years)	Autism (DSM-IV)	ADOS, ADI-R	Inclusion criteria in patients with autism: Clinical diagnosis of Autism according to DSM-IV and confirmed by ADI-R and ADOS-G	developmental delay TD	13 (54%) 15 (73%)	4.29 years (2.69–7.11 years) 3.69 years (1.35–5.84 years)	Overnight PSG with 21 lead electroencephalogram montage	CSHQ
Tessier, 2015, Canada [33]	13 (100%)	10.23 years (6–13 years)	hfASD(DSM-IV-TR)	ADI, ADOS	Inclusion criteria in patients with autism: Diagnosis based on ADOS and ADI-R and confirmed by DSM-IV criteria Exclusion criteria in patients with autism: Sleep disturbances (sleep apnea, PLMS) Atypical EEG patterns Medications (except for 3 children who were treated with methylphenidate) FSIQ < 75	TD	13 (100%)	10.23 years (7–12 years)	Recordings took place on 2 consecutive nights in individual bedrooms using bilateral central, frontal and occipital EEG leads (C3, C4, F3, F4, O1, O2)	Sleep diary
Tessier, 2015 Canada [34]	13 (100%)	10.23 years (6–13 years)	hfASD(DSM-IV-TR)	ADI-R, ADOS	Inclusion criteria in patients with autism: Diagnosis based on ADOS and ADI-R and confirmed by DSM-IV criteria Exclusion criteria in patients with autism: Sleep disturbances (sleep apnea, PLMS) Atypical EEG patterns Medications (except for 3 children who were treated with methylphenidate) FSIQ < 75	TD	13 (100%)	10.23 years (7–12 years)	Recordings took place on 2 consecutive nights in individual bedrooms using bilateral central, occipital and parietal EEG leads (C3, C4, O1, O2, P7, P8)	Sleep diary
Lambert, 2015 Canada [35]	11 (100%)	10.27 years (6–13 years)	hfASD(DSM-IV)	ADI-R, ADOS	Inclusion criteria in patients with autism: Diagnosis based on ADOS and ADI-R and confirmed by DSM-IV criteria Exclusion criteria in patients with autism: Diagnosis of other axis-I disorders Medical disorders other than autism Taking medications Sleep problems FSIQ < 70	TD	13 (100%)	10.23 years (7–12 years)	Participants spent two consecutive nights in the sleep laboratory, the first night served for adaptation to recording conditions. All PSG data reported were recorded during the second night. The electrodes that have been used are not specified	CSHQ
Sahroni, 2015, Japan [36]	8 (87.5%)	10.23 years (7–12 years)	Autistic disorder (DSM-IV)		Inclusion criteria in patients with autism: Diagnosis based on DSM-IV criteria Exclusion criteria in patients with autism: Presence of illness Taking medications	TD	8 (62.5%)	6.14 ± 2.19 years	Both groups were given some sedative before EEG recordings to make subjects sleep in a short time of period (10–15 min) 19 electrodes were placed according to the 10–20 international system	___
Maski, 2015, USA [37]	22 (86%)	11.3 years (9–16 years)	ASD (DSM-IV)	ADOS, ADI R	Inclusion criteria in ASD group: Diagnosis based on the ADOS and ADI-R NVIQ not more than 1.33 standard deviations below the mean Exclusion criteria in all participants: Significant hearing or vision loss Metabolic disease, neurogenetic disorders or co-occurring neurological disorders A previously diagnosed sleep disorder An unstable chronic medical condition such as asthma, diabetes, cystic fibrosis or cardiac disease Exclusion criteria in TD group: Sleep disorder, psychiatric disorder or neurodevelopmental condition Use of medications known to affect sleep, memory or daytime vigilance	TD	20 (90%)	12.3 years (9–16 years)	Home PSG recordings with seven channels of EEG (F1, F2, C3, Cz, C4, O1, O2)	CSHQ
Lehoux, 2017, Canada [38]	13 (100%)	10.23 years (6–13 years)	hfASD(DSM-IV)	ADI R	Inclusion criteria in ASD patients: Diagnosis based on ADOS and ADI-R and confirmed by DSM-IV criteria Exclusion criteria in ASD patients: Taking medications interacting with the central nervous system Intellectual disability Reported poor sleep Presence of other diagnosis besides ASD	TD	13	10.23 years (7–12 years)	PSG montage included 7 EEG channels (F3, F4, C3, P3, P4, O1, O2).	Sleep diary
Aathira, 2017, India [39]	71(90.3%)	5.3 ± 1.8 years (3–10 years)	ASD (DSM-IV)		Inclusion criteria in ASD group: Diagnosis based on DSM-IV criteria Exclusion criteria in both groups: Chronic systemic disorders known to interfere with sleep Exclusion criteria in ASD group: Presence of tuberous sclerosis, fragile X syndrome and Down syndrome	TD	65 (61.5%)	5.7 ± 1.6 years (3–10 years)	Single overnight PSG	CSHQ
Vite, 2018, Messico [40]	10 (100%)	8.2 years (6–10 years)	ASD, level 1 (DSM-IV and DSM-V)		Inclusion criteria in ASD group: Having a multiaxial diagnosis of ASD according to DSM-IV and DSM-V criteria Exclusion criteria in TD group: Presence of sleep disturbances Taking medications Previous diagnosis of a chronic disease Health issues that modifysleep at the time of registration Failure to complete 2 nights of PSG Exclusion criteria in ASD group: Failure to complete 2 nights of PSG	TD	7 (100%)	8.3 years (6–10 years)	2 eight-hour PSG were performed for 2 consecutive nights. The referrals for night 1 were: C3, C4, O2, O1 The referrals for night 2 were: F3, F4, C3, C4, T3, T4, P3, P4, O1, O2	-
Farmer, 2018 USA [41]	85 (84%)	2–6 years	Autistic disorder (DSM-IV-TR)	ADI, ADOS	Inclusion criteria in patients with autism: Diagnosis based on ADOS and ADI-R and confirmed by DSM-IV criteria Inclusion criteria in DD group: Cognitive score at least 1.5 SDs below the mean No diagnosis of ASD Inclusion criteria in TD group: No evidence of personal or first-degree family history of autism or significant DD	TD DD	29 (72%) 21 (62%)	2–6 years 2–6 years	Overnight video-EEG	-
Page, 2019, USA [42]	7 (71.4%)	21.8 months (13–30 months)	ASD (DSM-5)	FYI, M-CHAT-R/F, ADOS-2	Inclusion criteria in ASD group: High risk for ASD screened with the FYI and the M-CHAT-R/F and confirmed with the ADOS-2 Inclusion criteria in both groups: Sleeping between 8 and 14 hr at night One nap per day Free of known sleep disorder Free of medications Exclusion criteria in both groups: Reported history of presence of epilepsy Neurological/metabolic or developmental disability other than ASD Severe visual or motor impairment	TD	13 (38.4%)	21.8 months (13–30 months)	EEG was recorded during a daytime nap (average duration of 78 min) with a 124- or 128-channel high density EEG electrode net	Sleep diary
Fletcher, 2019, UK [43]	20 (80%)	125.55 months	autism	GARS	Inclusion criteria in ASD group: GARS-AI scores ≥ 71 Exclusion criteria in ASD group: Diagnosis of co-occurring conditions (i.e., dyslexia) Inclusion criteria in TD group: Not a sibling of a child with ASD GARS-AI < 55 No diagnosed psychological disorder	TD	34 (50%)	118.94 months	Home PSG with a montage of six EEG (F3, F4, C3, C4, O1, O2)	CSHQ
Arazi, 2019, Israel [44]	29 (72.4%)	4.6 years (1.9–7.8 years)	autism (DSM-5)	ADOS	Inclusion criteria in ASD group: Diagnosis based on ADOS and confirmed by DSM-V criteria 5 children with ASD were excluded from the final analysis due to poor PSG evaluationExclusion criteria in TD group: Snoring Sleep problems such as bedwetting, daytime sleepiness Neurological, psychiatric or developmental disorders	TD	23 (65.2%)	5.3 ± 1.5 years	PSG with 6 EEG electrodes (C3, C4, O1, O2, A1, A2)	CSHQ

AD: Autistic Disorders; ADI-R: Autism Diagnostic Interview–Revised; ADOS: Autism Diagnostic Observation Schedule; ADOS-2: Autism Diagnostic Observation–2nd Edition; ADOS-G: Autism Diagnostic Observation Schedule–Generic; AS: Asperger syndrome; ASD: autism spectrum disorder; CARS: Childhood Autism Rating Scale; CSHQ: Children’s Sleep Habits Questionnaire; DD: developmental delay; DSM-IV: Diagnostic and Statistical Manual of Mental Disorders, 4th edition; DSM-5: Diagnostic and statistical manual of mental disorders (5th ed.); EEG: electroencephalogram; FSIQ: full-scale Intelligence Quotient; FYI: first year inventory; GARS: Gilliam Autism Rating Scale; gsASD: good sleepers ASD; hfASD: high functioning ASD; *ICD-10: International Statistical Classification of Diseases and Related Health Problems, 10th revision*; IQ: Intelligence Quotient; M-CHAT-R/F: Modified checklist for autism in toddlers, revised with follow-up; MRI: magnetic resonance imaging; NVIQ: Non Verbal Intelligence Quotient; NRegASD: not regressive ASD; PCQ: Parental Concerns Questionnaire; PDD-NOS: pervasive developmental disorder not otherwise specified; PDSS: pediatric daytime sleepiness scale; PIQ: Performance Intelligence Quotient; PLMS: periodic leg movement syndrome; psASD: poor sleepers ASD; PSG: polysomnography; RegASD: regressive ASD; TD: typically-developing.

**Table 2:** Main significant findings, with *p*-value in brackets.

First Author, Year, Country	Macrostructural EEG Features	Microstructural EEG Features	Sleep Subjective Features	Other Findings
Elia, 2000, Italy [25]	ASD vs. TD ↓ TIB (*p* < 0.01), TST (*p* < 0.02), SPT (*p* < 0.01) ASD vs. X-fragile ↓ SPT (*p* < 0.03), RL (*p* < 0.01), N1 (*p* < 0.05)			
Malow, 2006, USA [26]	psASD vs. gsASD 1st night ↓ SE (*p* = 0.0091), REM% (*p* = 0.0226) ↑ SL (*p* < 0.0079), N3,N4 (*p* = 0.446) 2nd night ↓ TST (*p*= 0.3800) gsASD vs. TD 1st night ↓ TST (*p*= 0.5507) 2nd night ↓ TST (*p*= 0. 5483)			
Miano, 2007, Italy [27]	ASD vs. TD ↓ TIB (*p* < 0.044), SPT (*p* < 0.007), RL (*p* < 0.02)	ASD vs. TD ↓ CAP rate during N3,4 (*p* < 0.02) A1% (*p* < 0.0004) ↑ A2% (*p* < 0.006) A3% (*p* < 0.02)	ASD vs. TD Sleep Questionnaire: ↑ Sleep less than 8 hours (*p* < 0.02) Latency to sleep > 30 min (*p* < 0.000001) Difficulty falling asleep at night (*p* < 0.002) Fluids or drugs to facilitate sleep (*p* < 0.00001) Hypnic jerks (*p* < 0.00001) Rhythmic movements while falling asleep (*p* < 0.00001) Poor sleep quality (*p* < 0.00001) More than two awakenings per night (*p* < 0.05) Waking up to drink or to eat at night (*p* < 0.015) Difficulty to fall asleep after awakenings (*p* < 0.00001) Parasomnias – bedwetting (*p* < 0.00001) Daytime somnolence (*p* < 0.03) Falling asleep at school (*p* < 0.02) ↓ Drinks stimulant beverages in the evening (*p* < 0.00001)	
Bruni, 2007, Italy [28]	No significant results	AS vs. TD ↑ A1% (η^2^ = 1.43; *p* < 0.009) ↓ A2% (η^2^ = −1.88; *p* < 0.003) AS vs. ASD ↑ CAP rate during N3,4 (η^2^ = 1.41; *p* < 0.02) A1% (η^2^ = 2.05; *p* < 0.001)	AS Sleep Questionnaires: reluctant to go to bed (50%) need for light or TV in the bedroom (75%) difficulty getting to sleep at night (87%) falling asleep sweating (75%) nocturnal hyperkinesia(50%) feeling unrefreshed upon morning awakening (50%) difficulty in waking up in the morning (87%) daytime somnolence 87%) PDSS mean score 16.5 ± 3.4	AS Positive correlation between verbal IQ and: total CAP rate (r = 0.99) CAP rate in SWS (r = 0.95) global A1 index (r = 0.94) SWS A1 index (r = 0.76) Negative correlation between A2% and: FSIQ (r = −0.086) VIQ (r = −0.86) PIQ (r = −0.81) Positive correlation between CBCL total score and: cap rate (r = 0.76) A1 index (r = 0.88) Negative correlation between externalizing score and A3% (r= −0.81)
Goldman, 2009, USA [29]	psASD vs. TD ↑ SL (*p* < 0.05) psASD vs. gsASD ↑ SL (*p* < 0.05)		PCQ: poor sleepers rate among ASD: 64% psASD vs. gsASD CSHQ: ↑ sleep onset delay (*p* < 0.01), sleep duration (*p* < 0.01), night wakings and total (*p* < 0.01) psASD vs. TD ↑ for all dimensions except sleep disordered breathing	
Ming, 2009, USA [30]	ASD vs. TD ↓ REM% (*p* = 0.002)		ASD Sleep Questionnaires: Parasomnias (60.8%), Disorder of Partial Arousal (55.6%)	
Giannotti, 2010, Italy [31]	NregASD vs. RegASD ↑ TST (*p* < 0.001), SE (*p* < 0.001) ↓ WASO (*p* < 0.001), SL (*p* < 0.001) RegASD vs. TD ↓ TST (*p* < 0.001), SE (*p* < 0.001), REM% (*p* < 0.01), N3,4 (*p* < 0.001) ↑ WASO (*p* < 0.001), SL (*p* < 0.001), RL (*p* < 0.01), N2 (*p* < 0.001) NRegASD vs. TD ↓ TST (*p* < 0.001), SE (*p* < 0.001) ↑ WASO (*p* < 0.001), SL (*p* < 0.001), RL (*p* < 0.01)	ASD vs. TD ↓A1% (*p* < 0.001) ↑A2% (*p* < 0.01) A3% (*p* < 0.001) RegASD vs. TD ↓CAP rate during N1,2 (*p* < 0.01)	NregASD vs. RegASD CSHQ: ↓ Bedtime, Bedtime resistance, Sleep onset delay, Sleep duration, Night-wakings (*p* < 0.001); Sleep latency (*p* < 0.05) ↑ Sleep length (*p* < 0.001) RegASD/NRegASD vs. TD ↓ Sleep length, (*p* < 0.001) ↑ Bedtime, Sleep latency, Bedtime resistance, Sleep onset delay, Sleep duration, Night-wakings (*p* < 0.001)	
Buckley, 2010, USA [32]	ASD vs. TD ↓TST (*p* = 0.004), REM% (*p* < 0.001) ↑RL (*p* = 0.016), N3,4 (*p* = 0.001) ASD vs. DD ↓TST (*p* = 0.001), REM% (*p* < 0.001) ↑RL (*p* = 0.012), N3,4 (*p* < 0.001)		CSHQ: Median wake time: ASD 06.17 DD 06.45 TD 06.46	
Tessier, 2015, Canada [33]		hfASD vs. TD Fp1 ↓Sleep Spindles duration (*p* < 0.05) Fp2 ↓Sleep Spindles density (*p* < 0.05) ↓Fast sigma EEG activity at C3, C4 (*p* < 0.05)	Sleep diary: No sleep disturbances complained in the previous 14 days.	TD negative correlation between VIQ and Fp2 spindle density for the last quarter of the night (r= −0.6, *p* < 0.04) positive correlation: between VIQ and C4 spindle duration for the total night (r = 0.72, *p* = 0.01) between PIQ and fast sigma activity in the end of the night at the C4 electrode (r = 0.59, *p* = 0.04) ASD negative correlation: between VIQ and C3 spindle density for the total night (r= −0.62, *p* = 0.02) between FSIQ and C3 spindle density for the total night (r= −0.55, *p* = 0.05)
Tessier, 2015 Canada [34]	No significant results		Sleep diary: No sleep disturbances complained in the previous 14 days.	ASD vs. TD ↑ neutral emotion reaction times on the delayed recognition task ((η^2^ = 0.16, *p* = 0.04)
Lambert, 2015 Canada [35]	ASD vs. TD ↑SL (*p* = 0.02) ↓N3,4 (*p* = 0.026)	ASD vs. TD Fp1 ↓K-complex (*p* = 0.006) Fp2 ↓Sleep Spindles density (*p* = 0.03), K-complex (*p* = 0.013) C3 ↓K-complex (*p* = 0.002) C4 ↓K-complex (*p* = 0.006)	ASD vs. TD CSHQ: No significant results. Agendas: ↓Sleep onset latency (*p* < 0.05) Sleep quality (*p* < 0.02)	ASD Negative correlation between N1% and FSIQ (r = −0.53, *p* = 0.009) and PIQ (r = −0.65, *p* = 0.001) Negative correlation between N3,4% and CBCL internalized behaviors (r = −0.41, *p* = 0.046). Positive correlation between SL reported in daily sleep agendas and in PSG in both groups (r = 0.75, *p* < 0.001).
Sahroni, 2015, Japan [36]		ASD vs. TD ↑ absolute theta band power in T6 (*p* = 0.0379) ↑ absolute alpha band power in F7, Fz, F4, T3, Cz, C4, P3 (*p* < 0.03) ↑ relative delta band power in Fz, T6 (*p* = 0.0379) ↓ relative beta band power in T6 (*p* < 0.04) ↓ absolute and relative gamma band power in Fp1, T5, P3, T6, O1, O2 (*p* < 0.04)		
Maski, 2015, USA [37]	ASD vs. TD ↑ TIB (*p* = 0.01), WASO (*p* = 0.02), SL (*p* = 0.01) ↓ SE (*p* < 0.001), REM% (*p* = 0.007)		ASD vs. TD CSHQ: ↑ Bedtime resistance (*p* = 0.03), Sleep onset delay (*p* = 0.02), Sleep duration (*p* = 0.04), Sleep anxiety (*p* = 0.001), Daytime sleepiness (*p* < 0.02), Parasomnias (*p* = 0.02)	ASD vs. TD No significant differences in benefiting from sleep in memory consolidation tasks
Lehoux, 2017, Canada [38]	ASD vs. TD ↓N3,4 (*p* = 0.007)		Sleep diary: No sleep disturbances complained in the previous 14 days.	
Aathira, 2017, India [39]			poor sleepers rate among ASD: 77.5% ASD vs. TD CSHQ: ↑ Daytime sleepiness (*p* < 0.001), Parasomnias (*p* < 0.001), Sleep anxiety (*p* = 0.002), Bedtime resistance (*p* < 0.001)	psASD vs. gsASD ↑ higher CBCL mean score (*p* = 0.004), CBCL “withdrawn” score in the borderline or clinical range (*p* = 0.03) Not significant results about IQ and CARS
Vite, 2018, Messico [40]		ASD vs. TD ↑ Mu rhythm peak in C3 (*p* = 0.003) ↓ Mu rhythm peak in C4 (*p* = 0.003)		
Farmer, 2018 USA [41]		ASD vs. TD ↓Sleep Spindles density (*p* < 0.0001) ↓Sleep Spindles duration (*p* = 0.006) ASD vs. DD ↓Sleep Spindles density (*p* = 0.017)		For the full sample, significant correlation between: spindle density and IQ (r = 0.26, *p* < 0.002) spindle density and Vineland subscales: socialization (r = 0.33, *p* = 0.0001) communication (r = 0.32, *p* = 0.0002) living skills (r = 0.25, *p* = 0.003)
Page, 2019, USA [42]		ASD vs. TD ↓ theta band power in temporo-central regions (*p* < 0.05) ↑ beta band power in right temporo-occipital region (*p* < 0.05) ↑ slower sigma power over occipital and central regions (*p* < 0.05) ↓ higher frequency sigma power over frontal, central, and parietal regions (*p* < 0.05)	ASD vs. TD Sleep diary: No significant differences in the naptime nor in the duration of wakefulness before the nap.	No significant correlation between ADOS-2 score and NREM spectral power
Fletcher, 2019, UK [43]	ASD vs. TD ↓ TST (*p*≤0.05), NREM (*p* < 0.05)	ASD vs. TD ↓ sigma power(*p* ≤ 0.001)	ASD vs. TD CSHQ: ↑Total sleep problems (*p* < 0.001)	ASD vs. TD ↓performances in specific memory tasks with memory recalling after a month
Arazi, 2019, Israel [44]	ASD vs. TD ↓ TIB (*p* = 0.02), TST (*p* = 0.03), REM% - second half of the night (*p* = 0.007)		ASD vs. TD CSHQ: 50% of ASD children had scores that were above the mean score from previously published CSHQ scores from a large population of typically developing children in all domains, excluding sleep duration and sleep disordered breathing	Negative correlation between SWA power and Bedtime resistance (r= −0.49, *p* = 0.01), Total sleep disturbances (r= −0.38, *p* = 0.05) and time to fall asleep (r = 0.42, *p* = 0.02).

↓ Reduced value; ↑ Increased value; A1: subtype 1 of phase A of CAP; A2: subtype 2 of phase A of CAP; A3: subtype 3 of phase A of CAP; ADOS-2: Autism Diagnostic Observation Schedule-2nd Edition; AS: Asperger syndrome; ASD: autism spectrum disorder; CAP: cyclic alternating patterns; CARS: Childhood Autism Rating Scale; CBCL: Child Behavior Checklist; CSHQ: Children’s Sleep Habits Questionnaire; DD: developmental delay; FSIQ: Full Scale Intelligence Quotient; gsASD: good sleepers ASD; hfASD: high functioning ASD; IQ: Intelligence Quotient; N1: stage 1 of NREM; N2: stage 2 of NREM; N3: stage 3 of NREM; N4: stage 4 of NREM; NRegASD: not regressive ASD; NREM: non-rapid eye movement; PIQ: Performance Intelligence Quotient; psASD: poor sleepers ASD; PCQ: Parental Concerns Questionnaire; PSG: polysomnography; r: correlation coefficient; RegASD: regressive ASD; REM: rapid eye movement; RL: REM latency; SE: sleep efficiency; SL: sleep latency; SPT: sleep period time; SWA: slow wave activity; SWS: slow wave sleep; TD: typically developing children; TIB: time in bed; TST: total sleep time; VIQ: Verbal Intelligence Quotient; WASO: wakefulness after sleep onset; η^2^: Effect size.

## Supplementary Materials

The following are available online at https://www.mdpi.com/article/10.3390/jcm10173893/s1., Table S1: Data about macrostructural findings, Table S2: Data about microstructural findings, Table S3: CSHQ’s subscales and total score: comparisons between ASD and TD, Table S4: Summary comparison table.
