# Supplementary material for: Subjective and Electroencephalographic Sleep Parameters in Children and Adolescents with Autism Spectrum Disorder: A Systematic Review
Source: J Clin Med. 2021 Aug 30;10(17):3893. doi: 10.3390/jcm10173893 (PMC8432113; doi:10.3390/jcm10173893)
Supplement: Supplementary file 1 [file jcm-10-03893-s001.zip › jcm-1314056-supplementary.pdf]

Table S1. Data about macrostructural findings.

Author Year	Sample	TIB (minutes)*	TST	SPT	SE (%)	WASO**	SL (minutes)	RL (minutes)	REM%	NREM (%)
Elia, 2000 [25]	X fragile	518.00 ± 20.75	485.21 ± 11.94	500.71 ± 15.99	94±4	3.1±3.08		156.64±54.41	17.72±4.9	N1 = 4.84 ± 1.36<br>N2 = 49.09 ± 3.11<br>N3, N4 = 24.95 ± 2.62
	ASD	504.12 ± 26.89	448.62 ± 49.92	463.15 ± 45.01	89±7	3.1±5.53		91.38±37.49	19.43±4.73	N1 = 4.69 ± 1.99<br>N2 = 45.13 ± 3.74<br>N3, N4 = 27.53 ± 5.20
	TD	552.60 ±33.65	523.50 ± 23.84	536.80 ± 26.77	95±3	2.45±2.04		98.6±26.88	22.58±1.36	N1 = 5.92 ± 2.33<br>N2 = 46.82 ± 2.48<br>N3, N4 = 22.25 ± 3.65
	ASD poor sleepers		434.1 ± 66.5		75.8±9.3	29.9±19.2	97.8±71.2	162.2±54.2	12.5±5.4	N1 = 9.1±3.4<br>N2 = 45.8±7.1<br>N3, N4 = 32.6±8.1
1st night	ASD good sleepers		462.6 ± 68.7		87.5±9.0	29.9±27.1	30.3±17.7	144.3±40.8	17.1±3.3	N1 = 11.6±3.8<br>N2 = 46.2±7.6<br>N3, N4 = 25.1±6.5
Malow 2006 [26]	TD		469.8 ± 28.7		86.7±4.1	45.7±28.4	25.1±19.4	171.8±73.9	16.2±3.9	N1 = 11.2±6.1<br>N2 = 48.5±4.2<br>N3, N4 = 24.1±3.9
	ASD poor sleepers		465.8 ± 48.2		85±6.5	24±13.9	57.1±50	162±26.2	16.6±3.2	N1 = 8.4±4.4<br>N2 = 47.4±8.1<br>N3, N4 = 27.7±9.3
2nd night	ASD good sleepers		475.5 ± 62.8		88.2±7.5	21±6.7	29.4±38.6	148.9±61.3	17.8±4.9	N1 = 10.2±5.6<br>N2 = 45.1±7.2<br>N3, N4 = 27.0±7.3
	TD		484.9 ± 31.7		87.7±4.1	32.3±13.1	33.6±22.1	150.7±65.2	16.8±2.8	N1 = 8.9±3.5<br>N2 = 46.6±5.0<br>N3, N4 = 27.8±4.3
	ASD	492.9 ± 36.23	438.5 ± 54.94	453.9 ± 53.94	89±8.67	3.3±5.31	37.5±42.18	84.3±39.27	21.9±3.95	N1 = 5.8 ± 7.35<br>N2 = 43.2 ± 10.88<br>SWS = 25.9 ± 10.38
	TD	534.3 ±52.98	493 ± 46.87	505.5 ± 47.65	92.5±5.37	2.4±3	22.9±14.58	114.6±37.16	23.3±5.1	N1 = 4 ± 4.31

Miano, 2007 [27]										N2 = 47.1 ± 5.47<br>SWS = 23.3 ± 5.48
Bruni, 2007 [28]	Autism	485.3 ± 39.47	425.8 ± 57.68	444.6 ± 54.87	87.9	4.1	39.2±47.85	78.4	22.1	N1 = 6.5 ± 8.48<br>N2 = 41.5 ± 13.50<br>SWS = 25.8 ± 12.86
	Asperger	528.4 ± 61.76	452.2 ± 50.28	484.4 ± 62.72	86	6.1	28.86±29.46	112.2	20.3	N1 = 5.1 ± 4.09<br>N2 = 42.3 ± 9.63<br>SWS = 26.2 ± 8.79
	TD	529.2 ± 59.58	489.8 ± 48.53	508.1 ± 52.38	92.8	3.5	19.6±14.25	106.6	24.1	N1 = 4.2 ± 4.91<br>N2 = 46.1 ± 4.57<br>SWS= 22.0 ± 6.24
Goldman, 2009 [29]	ASD poor sleepers		495.9 ± 65.8		84.7±7	25.7±14.9	54.0±41.7			
	ASD good sleepers		489.3 ± 52.4		88.3±6.2	26.2±23.1	31.1±28.9			
	TD		488.4 ± 44.7		87.8±6.1	29.1±18.6	34.9±34.3			
Ming, 2009 [30]	ASD		395 ± 94				16.8±22	146±106	14.2±7.7	
	TD		451 ± 59				22.4±13.9	114±47	20.4±4.6	
Giannotti, 2011 [31]	ASD without regression		525.20 ± 52.73		84.18±6.95	9.55±5.43	35.25±18.77	154.02±97.74	14.7±6.56	N1 = 9.12 ± 4.74<br>N2 = 47.65 ± 5.47<br>SWS = 19.35 ± 4.93
	ASD with regression		451.30 ± 58.30		74.55±8.56	15.9±9.31	53.1±24.6	198.91±101.51	7.75±5.62	N1 = 8.96 ± 9.57<br>N2 = 54.50 ± 8.02<br>SWS = 12.89 ± 3.11
	TD		610.20 ± 66.38		94.7±2.77	2.1±2.7	20.4±5.47	99.65±21.21	22.68±1.6	N1 = 7.25 ± 1.08<br>N2 = 41.30 ± 2.38<br>SWS = 26.67 ± 2.03
Buckley, 2010* [32]	Autism		7.59 ± 1.66		83.7±16.5	50.1±82.7	28.5±65.5	108.5±80.3	14.5±8.4	N1 = 4.7 ± 4.9<br>N2 = 56.4 ± 12.3<br>SWS = 21.5 ± 9.0
	Developmental delayed		9.01 ± 0.88		87.8±8.9	45.1±43.8	33±11.5	69±27.5	25±6.8	N1 = 2.1 ± 2.3<br>N2 = 57.3 ± 10.7<br>SWS = 13.7 ± 7.1

	TD		8.81 ± 0.93	86.2±11.8	37±40	37.5±34.5	64±59	22.6±6.5	N1 = 3.7 ± 1.2<br>N2 = 55.8 ± 8.5<br>SWS = 18.6 ± 5.4
Lambert, 2016 [34]	Autism	*Weekdays*<br>9.77 ± 0.47<br>*Weekends*<br>9.74 ± 0.61	ASD: 9.78 ± 0.37	97.08±1.42	23.77±7.96	32.97±27.69	106.0±37.7	17.05±3.96	N1 = 7.82 ± 3.28<br>N2 = 56.89 ± 5.34<br>SWS = 18.24 ± 3.15
	TD	*Weekdays*<br>9.83 ± 0.59<br>*Weekends*<br>9.74 ± 0.61	9.92 ± 0.51	97.22±1.17	24.73±11.25	14.44±16.58	126.13±50.21	16.4±3.08	N1 = 6.06 ± 2.10<br>N2 = 53.91 ± 6.27<br>SWS = 23.64 ± 5.70
Maski, 2015 [35]	ASD	583 ± 17	469 ± 14	86±2	45.9 (8.1, 172.1)	27.3 (5, 101)		18.9±1.6	N1 = 4.1<br>N2 = 47.3<br>SWS = 29.7
	TD	506 ± 17	500 ± 16	93±1	21.5 (8.8, 51.5)	11 (0, 39)		22.9±1.1	N1 = 3.2<br>N2 = 44.4<br>SWS = 29.5
Lehoux, 2017 [36]	ASD		568.79 ± 53.46	97.31±1.46	24.3±11.26	30.43±26.11	107.1±35.72	17.13±3.67	N1 = 7.54 ± 3.14<br>N2 = 56.96 ± 4.88<br>SWS = 18.37 ± 2.90
	TD		539.67 ± 60.21	97.22±1.17	23.77±7.96	14.4±16.58	126.13±50.21	16.4±3.08	N1 = 6.06 ± 2.10<br>N2 = 53.91 ± 6.27<br>SWS= 23.64 ± 5.70
Aathira, 2017 [45]	ASD		<8 h: 95.8%	<95%:70.8 %					<150 min: 75%
Fletcher, 2019 [37]	ASD		504.88±36.03		34±34.4				N1 = 19.85 ± 10.97<br>N2 = 240.69 ± 46.26<br>N3 = 120.85 ± 31.27<br>NREM = 361.54 ± 34.38
	TD		546.48±37.11		24.88±16.64				N1 = 26.46 ± 18.09<br>N2 = 247.18 ± 39.91

										N3 = 140.58 ± 27.77<br>NREM = 387.76 ± 28.43
Arazi, 2019 [38]	ASD	391 ± 70	362 ± 72		92.5±7	6.9±10.1	7.5±8.4		*1st half of night*<br>10.6±5.5<br>*2nd half of night*<br>19.1±8.5	*1st half of night*<br>N2 49.8 ± 12.5<br>N3 39.4 ± 12.4<br>*2nd half of night*<br>N2 65.8 ± 9.6<br>N3 15.2 ± 10.6<br>*Whole night*<br>N2 57.8 ± 8.7<br>N3 27.1 ± 9.1
	TD	427 ± 40	403 ± 38		94.30±3	3.7±2.6	9.8±8.5		*1st half of night*<br>8.5±5.6<br>*2nd half of night*<br>25.6±8	*1st half of night*<br>N2 40.1 ± 8.8<br>N3 50.5 ± 10.1<br>*2nd half of night*<br>N2 64.2 ± 10.5<br>N3 10.1 ± 7.9<br>*Whole night*<br>N2 52.8 ± 7.3<br>N3 29.2 ± 5.2

Data are reported as mean±standard deviation or as median (min, max). *TIB is reported in hours by Lambert. **WASO is reported as % by Elia, Miano, Bruni, Giannotti, Arazi; as minutes by Goldman, Buckley, Lambert, Maski, Fletcher; as absolute number by Lehoux.

Table S2. Data about microstructural findings

Author Year	Sample	Total CAP rate	A1%	A2%	A3%	Spindle duration	Spindle density	Fast sigma activity	K-complex density
Miano, 2007 [27]	ASD	36.8 ± 10.39	65.1 ± 8.35	19.7 ± 6.23	15.1 ± 6.16				
	TD	37.9 ± 7.27	77.9 ± 8.43	12.8 ± 7.03	9.4 ± 3.02				
Bruni, 2007 [28]	Autism	37.02 ±8.39	64.90 ±9.22	21.05 ± 7.48	14.03 ± 6.21				
	Asperger	29.53 ± 15.52	80.24 ± 4.29	8.59 ± 4.45	11.14 ± 4.59				
	TD	42.54 ± 8.05	69.40 ± 9.05	19.08 ± 6.19	11.51 ± 5.23				
Giannotti, 2011 [31]	ASD without regression	31.28 ± 5.2	55.20 ± 4.33	24.24 ± 2.87	20.46 ± 5.10				
	ASD with regression	27.12 ± 4.60	45.65 ± 3.17	27.97 ± 1.21	26.38 ± 3.31				
	TD	34.45 ± 5.4	72.53 ± 2.04	14.07 ± 0.82	13.50 ± 1.78				
Tessier, 2015* [40]	Autism					0.85 ± 0.12	126.77 ± 87.09	3.81 ± 2.16	
	TD					0.96 ± 0.13	216.21 ± 121.16	8.12 ± 6.25	
Lambert, 2016* [34]	Autism						210.98 ± 113.30		53.09 ± 31.57
	TD						249.06 ± 132.12		125.80 ± 77.42
Farmer, 2018 [43]	Autism					1.27 ± 0.20	108.46 ± 45.71		
	TD					1.39 ± 0.25	153.59 ± 57.36		

Data are reported as mean±standard deviation; *these measures are calculated on the basis on statistical significant values provided by authors.

Table S3. CSHQ's subscales and total score: comparisons between ASD and TD. **CSHQ**

	Bedtime resistance		Sleep onset delay		Sleep duration		Sleep anxiety		Night wakings		Sleep disordered breathing		Daytime sleepiness		Parasomnias		Sleep total	
	ASD	TD	ASD	TD	ASD	TD	ASD	TD	ASD	TD	ASD	TD	ASD	TD	ASD	TD	ASD	TD
Malow [26]																		
(ASD poor sleepers)	9.60 (3.00)	7.20 (1.60)	2.20 (0.90)	1.30 (0.50)	6.10 (1.40)	4.10 (1.30)	7.60 (2.30)	5.70 (2.00)	5.50 (2.60)	4.00 (1.90)	4.27 (1.56)	3.20 (0.42)	15.45 (3.56)	12.60 (3.13)	10.64 (2.62)	8.30 (1.16)	57.64 (9.84)	42.10 (5.93)
(ASD good sleepers)	7.80 (2.60)	7.20 (1.60)	1.30 (0.50)	1.30 (0.50)	4.10 (1.30)	4.10 (1.30)	5.70 (2.00)	5.70 (2.00)	4.00 (1.90)	4.00 (1.90)	3.50 (0.53)	3.20 (0.42)	12.80 (4.29)	12.60 (3.13)	9.40 (2.27)	8.3 (1.16)	45.70 (8.08)	42.10 (5.93)
Goldman [29]																		
(ASD poor sleepers)	8.50 (2.90)	8.30 (2.70)	**2.10 (0.90)***	1.20 (0.50)	**5.70 (1.70)***	3.80 (1.10)	**6.50 (2.30)***	5.20 (2.00)	**5.50 (2.10)***	4.00 (1.20)	3.80 (1.20)	3.20 (0.4)	**15.40 (3.40)***	12.30 (3.00)	**10.50 (2.10) ***	8.60 (1.50)	**53.80 (6.70)***	43.80 (6.60)
(ASD good sleepers)	7.50 (2.30)	8.30 (2.70)	1.50 (0.60)	1.20 (0.50)	4.10 (1.40)	3.80 (1.10)	5.40 (1.80)	5.20 (2.00)	4.10 (1.80)	4.00 (1.20)	3.50 (0.70)	3.20 (0.40)	13.30 (3.70)	12.30 (3.00)	9.20 (2.00)	8.60 (1.50)	45.80 (8.20)	43.80 (6.60)
Giannotti [31]																		
(non regressed ASD)	**10.10 (3.42)****	7.20 (2.10)	**1.70 (0.92)****	1.00 (0.29)	**4.73 (2.35)****	3.10 (0.20)	4.90 (2.10)	4.68 (0.99)	**4.40 (1.76)****	3.10 (0.57)	3.31 (0.88)	3.20 (0.89)	10.10 (2.51)	9.92 (2.90)	8.10 (1.73)	7.90 (1.10)	/	/
(regressed ASD)	**13.80 (3.0)****	7.20 (2.10)	**2.40 (0.90)****	1.00 (0.29)	**6.50 (2.40)****	3.10 (0.20)	4.70 (2.89)	4.68 (0.99)	**6.90 (1.77)****	3.10 (0.57)	3.30 (0.99)	3.20 (0.89)	10.20 (3.90)	9.92 (2.90)	8.20 (2.50)	7.90 (1.10)	/	/
Lambert [34]	7.09 (1.92)	7.23 (2.20)	1.64 (0.92)	1.31 (0.63)	3.27 (0.65)	4.00 (2.24)	5.64 (1.69)	4.69 (1.70)	3.45 (0.69)	3.69 (0.75)	3.27 (0.47)	3.31 (0.869	12.18 (2.48)	11.31 (1.93)	8.18 (1.08)	7.92 (1.71)	42.09 (3.53)	41.15 (4.22)

Maski [35]	**7.8 (0.5)***	6.6 (0.2)	**1.9 (0.2)***	1.4 (0.1)	**4.9 (0.4)***	3.8 (0.3)	**6.1 (0.5)****	4.2 (0.1)	3.6 (0.2)	3.2 (0.1)	3.4 (0.2)	3.2 (0.1)	**14 (0.8)***	11.7 (0.4)	**9 (0.4)***	7.7 (0.2)	**49.0 ****	40.0
AAthira [45]	**13.6 (2.7)****	10.8 (2.8)	**2***	1	3.8 (1.8)	3.4 (1.0)	**8.1 (2.0)***	7.0 (2.1)	3.6 (1.2)	3.9 (1.7)	**3.3 (0.7)****	3.0 (1.1)	**10.0 (2.3)****	8.7 (2.0)	**8.5 (1.6)***	7.0 (1.5)	/	/
Fletcher [37]	/	/	/	/	/	/	/	/	/	/	/	/	/	/	/	/	**52.87** (9.76)**	40.94 (5.52)

*p<0.05; **P<0.001.

Table S4. Summary Comparison Table

ASD vs. TD	
**TIB**	
**Elia, 2000 [25]**	↓°
**Miano, 2007 [27]**	↓°
**Bruni, 2007 [28]**	↓
**Lambert, 2015 [34]**	weekdays↓<br>weekends↓
**Maski, 2015 [35]**	↑°
**Arazi, 2019 [38]**	↓°
**TST**	
**Elia, 2000 [25]**	↓°
**Malow, 2006 [26]**	↓
**Miano, 2007 [27]**	↓°
**Bruni, 2007 [28]**	↓
**Goldman, 2009 [29]**	Good sleepers ↑<br>Poor sleepers ↑
**Ming, 2009 [30]**	↓
**Giannotti, 2010 [31]**	Non regressive ↓°<br>Regressive ↓°
**Buckley, 2010 [32]**	↓°
**Lambert, 2015 [34]**	↓
**Maski, 2015 [35]**	↓
**Lehoux, 2018 [36]**	↑
**Fletcher, 2019 [37]**	↓°
**Arazi, 2019 [38]**	↓°
**SPT**	
**Elia, 2000 [25]**	↓°
**Miano, 2007 [27]**	↓°
**Bruni, 2007 [28]**	↓

Sleep efficiency	
**Elia, 2000 [25]**	↓
**Malow, 2006 [26]**	Good sleepers ↑<br>Poor sleepers ↓
**Miano, 2007 [27]**	↓
**Bruni, 2007 [28]**	↓
**Goldman, 2009 [29]**	Good sleepers ↑<br>Poor sleepers ↓
**Giannotti, 2011 [31]**	Non regressive ↓°<br>Regressive ↓°
**Buckley, 2010 [32]**	↓
**Lambert, 2016 [34]**	↓
**Maski, 2015 [35]**	↓°
**Lehoux, 2017 [36]**	↑
**Arazi, 2019 [38]**	↓
**WASO**	
**Elia, 2000 [25]**	↑
**Malow, 2006* [26]**	↓
**Miano, 2007 [27]**	↑
**Bruni, 2007 [28]**	↑
**Goldman, 2009 [29]**	↓
**Giannotti, 2010 [31]**	Non regressive↑ °<br>Regressive↑ °
**Buckley, 2010 [32]**	↑
**Lambert, 2015 [34]**	↓
**Maski, 2015 [35]**	↑°
**Lehoux, 2018 [36]**	↑
**Fletcher, 2019 [37]**	↑
**Arazi, 2019 [38]**	↑

SL

**Malow, 2006* [26]**	↓
**Miano, 2007 [27]**	↑
**Bruni, 2007 [28]**	↓
**Goldman, 2009 [29]**	ASD good sleeper ↓<br>ASD poor sleeper ↑°
**Ming, 2009 [30]**	↓
**Giannotti, 2010 [31]**	Non regressive↑ °<br>Regressive↑ °
**Buckley, 2010 [32]**	↑
**Lambert, 2015 [34]**	↑°
**Maski, 2015 [35]**	↑°
**Lehoux, 2018 [36]**	↑
**Arazi, 2019 [38]**	↓
**RL**	
**Elia, 2000 [25]**	↓
**Malow, 2006* [26]**	↓
**Miano, 2007 [27]**	↓°
**Bruni, 2007 [28]**	↑
**Ming, 2009 [30]**	↑
**Giannotti, 2010 [31]**	Non regressive↑ °<br>Regressive↑ °
**Buckley, 2010 [32]**	↑°
**Lambert, 2015 [34]**	↓
**Lehoux, 2018 [36]**	↓

**REM%**	
**Elia, 2000 [25]**	↓
**Malow, 2006* [26]**	↑
**Miano, 2007 [27]**	↓
**Bruni, 2007 [28]**	↓

**Ming, 2009 [30]**	↓°
**Giannotti, 2010 [31]**	Non regressive↓<br>Regressive↓ °
**Buckley, 2010 [32]**	↓°
**Lambert, 2015 [34]**	↑
**Maski, 2015 [35]**	↓°
**Lehoux, 2018 [36]**	↑
**Arazi, 2019 [38]**	↓° **
**N1 %**	
**Elia, 2000 [25]**	↓
**Malow, 2006 [26]**	↓ poor sleepers<br>↑ good sleepers *(2nd night)*
**Miano, 2006 [27]**	↑
**Buckley, 2010 [32]**	↑
**Giannotti, 2010 [31]**	↑ regressive ASD
**Lambert, 2015 [34]**	↑
**Maski, 2015 [35]**	↑
**Lehoux, 2017 [36]**	↑
**Fletcher, 2019 [37]**	↓
**N2%**	
**Elia, 2000 [25]**	↓
**Malow, 2006 [26]**	↓ poor sleepers *(1st night)*<br>↑ poor sleepers *(2nd night)*<br>↓ good sleepers
**Miano, 2006 [27]**	↓
**Buckley, 2010 [32]**	↑
**Giannotti, 2010 [31]**	↑° regressive ASD
**Lambert, 2015 [34]**	↑
**Maski, 2015 [35]**	↑
**Lehoux, 2017 [36]**	↑
**Fletcher, 2019 [37]**	↓

**Arazi, 2019 [38]**	↑ *(1st half of night)* °<br>↑ *(2nd half of night)*<br>↑ *(whole night)* °
**SWS%**	
**Elia, 2000 [25]**	↑
**Malow, 2006 [26]**	↑ poor sleepers *(1st night)* °<br>↑ good sleepers *(1st night)*
**Miano, 2006 [27]**	↑
**Buckley, 2010 [32]**	↑°
**Giannotti, 2010 [31]**	↓°
**Lambert, 2015 [34]**	↓°
**Maski, 2015 [35]**	↑
**Lehoux, 2017 [36]**	↓°
**Fletcher, 2019 [37]**	↓
**Arazi, 2019 [38]**	↓ *(1st half of night)* °<br>↑ *(2nd half of night)*<br>↓ *(whole night)*
